# Supplementary material for: Learning curve and surgical outcome of robotic assisted colorectal surgery with ERAS program
Source: Sci Rep. 2022 Nov 29;12:20566. doi: 10.1038/s41598-022-24665-w (PMC9709162; doi:10.1038/s41598-022-24665-w)
Supplement: Supplementary file 2 — Supplementary Information 2. [file 41598_2022_24665_MOESM2_ESM.zip › RAL Raw data-Ñ[▒K/068/OP_ñw╝╨░O▒Kñσ.pdf]

正式摘要(電子簽章：000000219845118)

請選擇...reporter

臺中榮民總醫院

Operation Note

[redacted]

- 手術日期：2021/06/16
- 手術醫師：VS 林俊余，CR 楊曙榮，R 張嘉育
- Anesthesia : GE
- Position : Lithotomy
- Aseptic agents : Soap , Betadine Alcohol , Betadine Aqua

Diagnosis

- Pre-operative diagnosis : s/p LAR with dehescience.
- Post-operative diagnosis : Ditto

Surgical Indication

- anastomosis dehescience

Operation

- Low anterior resection with colo-anal anastomosis
  Ileostomy

Finding

- 1. Anastomosis dehescience at lower rectum
  2. Severe adhision around pelvics and retroperitonium
  3. Take down of splenic flexure: yes
  4. Drain: two 12 mm penroe to pelvis.
  5. Hylobarrier +

Procedure

- After GA, the patient was placed in the modified Trendelenburg lithotomy position. The abdomen and perineum was prepped and draped in standard sterile fashion. A midline incision was made from above the umbilicus to the pubis. The abdomen was explored. The operative findings were mentioned as above. Using electrocautery, the colon was freed from its peritoneal attachments along the line of Toldt proximally from the descending colon and distally to the pelvic inlet. The peritoneum overlying the mesentery was then scored with electrocautery. The plane anterior to the aorta was entered and colon, mesenteric fat were mobilized from retroperitoneum overlying the sacral promontory and into the pelvis. The incision was extended laterally and medially across the peritoneal reflection. The mesorectum was then mobilized using sharp and blunt dissection. Care was taken not to enter the presacral venous plexus. The rectum was carefully elevated until an adequate distal margin was noted. The proximal colon was carefully inspected, noted to reach into the pelvis, and to have an excellent blood supply. The mesenteric fat was gently cleared from the terminal 2-3 mm of the bowel ends. The field was isolated with moist pad and the bowel opened and sized. The splenic flexure was taken down. The long-star anal retracter was extented the anus and the colon was suture with the rectum with 3-0 Vicryl.The abdomen cavity was then copiously irrigated and hemostasis was checked again and a closed suction was placed in the pelvis and brought out through separate stab wounds. The loop ileostomy was made over RLQ. The two penroce drain were put into pelvics. The wound was closured with #1 Vicryl and 3-0 Nylone.
- Estimated blood loss: minimum
- 器械數量: 正確
- 紗布數量: 正確

Image

- PAINT
    - abdomen

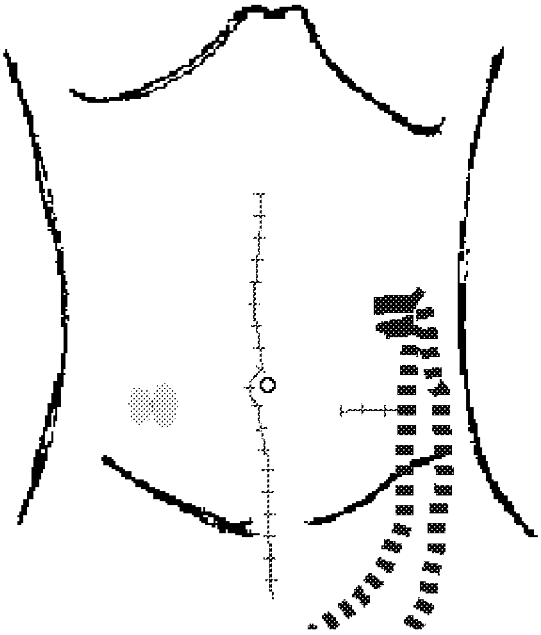

    - colon & rectum

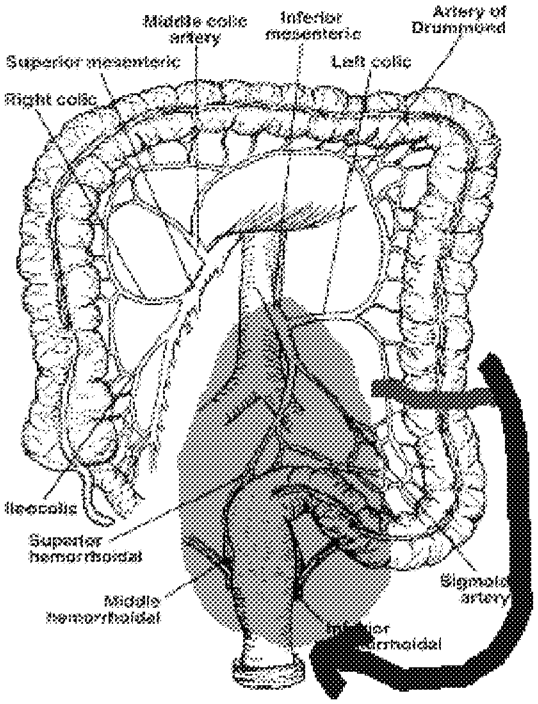

主治 2222H林俊余 / 醫師 2292G楊曙榮

正式摘要(**電子簽章：000000219845118)**

請選擇...reporter

doctorRank:B
